# Risk stratification by injury distribution in polytrauma patients – does the clavicular fracture play a role?

**DOI:** 10.1186/1754-9493-7-23

**Published:** 2013-07-04

**Authors:** Klemens Horst, Thomas Dienstknecht, Roman Pfeifer, Miguel Pishnamaz, Frank Hildebrand, Hans-Christoph Pape

**Affiliations:** 1 Department of Trauma and Reconstructive Surgery, University Hospital RWTH Aachen, Pauwelsstreet 30, 52074 Aachen, Germany

## Abstract

**Background:**

Thoracic and extremity injuries are common in polytraumatized patients. The clavicle limits the upper thoracic cage and connects the body and upper extremities. It is easy to examine and is visible on standard emergency room radiographs. We hypothesize that clavicular fracture in polytrauma patients indicates the presence of further injuries of the upper extremities, head, neck and thorax.

**Material and methods:**

Retrospective study including patients admitted between 2008 and 2012 to a level-I trauma center. Inclusion criteria: ISS > 16, two or more injured body regions, clavicular fracture. Control group: patients admitted in 2011, ISS > 16, two or more injured body regions, no clavicular fracture. Patient information was obtained from the patients’ charts; evaluation of radiographic findings was performed; scoring was based on the Abbreviated Injury Scale (AIS) and Injury Severity Score (ISS) AIS/ISS; data were analyzed using Pearson’s correlation and the Mann–Whitney *U*-test in SPSS (version 11.5.1); graphs were drawn using EXCEL®.

**Results:**

Thirty-four patients with clavicular fracture (C+) and 40 without (C-) were included; the mean ISS was 25 (range 16–57), m = 70%, f = 30%; age 43.3 years (range 9–88); clavicular fractures were positively correlated with severe thoracic (p = 0.011, OR 4.5: KI 1.3–15.3), external (p < 0.001, OR 9.2: KI 2.7–30.9) and upper extremity injuries (p < 0.001, OR 33.2: KI 6.9–16.04 resp. p = 0.004, OR 12.5: KI 1.5–102.9). C + showed a lower head/neck AIS (p = 0.033), higher thorax AIS (p = 0.04), arm/shoulder AIS (p = 0.001) and external AIS (0.003) than C-. Mean hospital stay and ICU treatment time were longer in the C + group (p = 0.001 and p = 0.025 respectively).

**Conclusion:**

A clavicular fracture can be diagnosed easily and may be used as a pointer for further thoracic and upper extremity injuries in polytrauma patients that might have been otherwise missed. Special attention should be paid on second and tertiary survey.

## Introduction

Accidents are the leading cause of death in children and young adults. In 2010 20,242 people died in Germany following a severe accident [1]. Management of seriously injured patients is highly demanding and interdisciplinary cooperation is necessary. In 2010 the German Society for Traumatology (DGU) published the first S3-Guideline to optimize management in polytraumatized patients [2]. Typically, the most severe injuries are found in the thoracic and abdominal area and in the long bones. Once the patient is brought to a Trauma Center, standardized algorithms of diagnostic procedures and treatment are performed. Due to the nature of polytraumatized patients, life-threatening injuries are the first priority. Overlooked and delayed diagnoses are common problems in the treatment of polytraumatized patients [3]. Buduhan et al. reported that 33.3% of upper extremity injuries were overlooked [4], while Kalemoglu et al. reported a rate of 38.2% [5]. In terms of wrist, hand and arm injuries, Guly reported rates of 17.2%, 21.7% and 15.1% of missed injuries, respectively [6]. Other authors reported that 4–8% (wrist/hand) and 11–12% (arm) of injuries of the upper extremities were missed. As thoracic injuries are common in polytrauma, taking plain thoracic x-rays during the emergency procedure is widely accepted. Beside typical thoracic injuries such as rip series fracture, hemopneumothorax or suspicious mediastinal signs, additional information such as the presence of clavicular fractures can be obtained from the x-ray. We hypothesize that a clavicular fracture in polytraumatized patients is suggestive of additional upper extremity injuries. Additionally we evaluate the circumstances that are responsible for the diagnosis of missed injuries in the upper extremity area and describe strategies to limit these pitfalls.

## Material and methods

This retrospective study included patients examined between 2008 and 2012. Inclusion criteria: clavicular fracture, ISS > 16 and injury to two or more physical regions or organ systems, where at least one injury was life threatening. This group was named C+. Inclusion criteria for the control group were the same except that these patients had no clavicular fracture. Consecutive patients that were diagnosed and treated in 2011 were included in the control group, named C-. Patient information was obtained from the patients’ charts and the hospital’s electronic database. Plain radiographs (chest and pelvic radiographs from primary survey; plain radiographs and CT scans of each injured region that were taken on day of admission or later during hospital stay) were evaluated by independent investigators (radiologists and trauma surgeons) and trauma severity was scored using the Abbreviated Injury Scale (AIS-90) and the Injury Severity Score. Statistical analysis was carried out using SPSS (version 11.5.1). Pearson’s correlation, the Mann–Whitney *U*-test and Chi-Square tests were used. A power analysis was performed using G*Power (version 3.1.5) and d = 0.8. Means and standard deviation are given. Statistical significance was defined as p = 0.05. Graphs were drawn using Microsoft EXCEL®.

## Results

**Figure 1:**
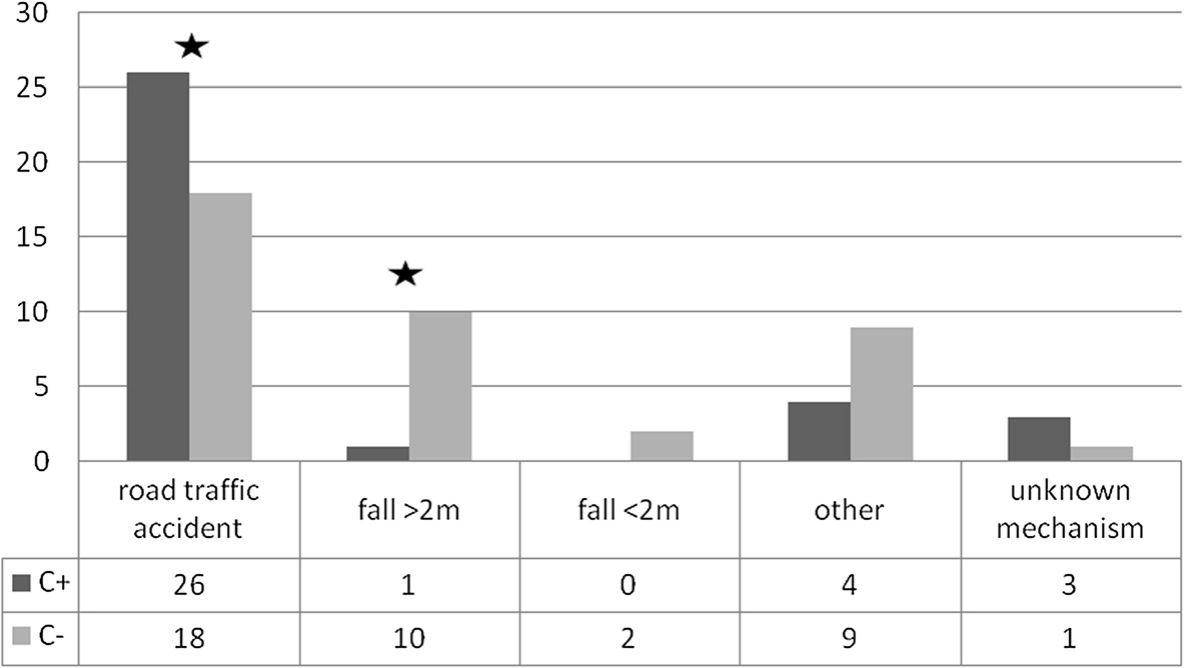
Trauma mechanisms, p < 0.05.

In total 74 patients met the inclusion criteria. Thirty-four patients had a clavicular fracture (age 46 ±20 years, m = 22, f = 12) and 40 patients had no clavicular fracture (age 41 ±19 years, m = 30, f = 10). Patients with a clavicular fracture were more frequently involved in road accidents (OR 3.97, KI 1.45–10.88, p = 0.006) than those without, and with the exception of falls from a height over two meters (OR 0.09, KI 0.01–0.75, p = 0.007), no significant associations with other trauma mechanisms were observed (Figure 1).

The mean hospital stay was 23 ±15 days in the C + group and 17 ±28 (p = 0.001) in the C- group. Patients with a clavicular fracture remained in the intensive care unit for 5 ±3 days, whereas patients without a fracture of the clavicle remained for 4 ±7 days (p = 0.025) days. The AIS for thoracic injuries as well as injuries of the arm/shoulder region and external injuries was higher in patients with fracture of the clavicle. The AIS for head injuries was higher in polytrauma patients without a clavicular fracture (Table 1). Five patients in the C + group died, three of them during emergency room procedures. Seven patients in the C- group died during hospital stay.

**Table 1:** Mean AIS in polytrauma patients with and without clavicular fracture

***Location***	***Group***	***AIS***	***SD***	***p***
*Head*	C+	1.24	1.76	0.033
	C-	2.20	2.00	
*Thorax*	C+	2.71	1.29	0.040
	C-	1.93	1.61	
*Arm/shoulder*	C+	1.94	0.55	0.001
	C-	0.50	0.82	
*External*	C+	0.97	0.46	0.003
	C-	0.58	0.71	

The ISS was higher in patients with clavicular fracture (25.79 ±7.03) than in patients without clavicular fracture (24.63 ±10.39; p = 0.047).

Correlation analysis of the AIS and ISS score of different body regions showed that there was a positive correlation between clavicular fracture and injuries of the thorax, arm/shoulder (AIS), extremities (ISS) and external injuries (AIS/ISS). Death was not correlated with the concomitant presence or absence of clavicular fracture (Table 2).

**Table 2:** Pearson’s correlation in polytrauma patients with and without clavicular fracture using AIS/ISS scoring regions

***Location***		***Clavicular fracture n%***		***Pearson’s correlation coefficient***	***p***
**AIS**	*Thorax*	32	40.5%	0.294	0.011
	*Arm/shoulder*	30	43.2%	0.629	>0.001
	*External*	32	40.5%	0.451	>0.001
**ISS**	*Chest*	32	40.5%	0.294	0.011
	*Extremities*	33	44.6%	0.332	0.004
	*External*	30	43.2%	0.565	>0.001
**Death**		5	6.8%	−0.038	0.749

The odds ratios of clavicular fracture and associated injures in AIS/ISS body regions are shown in Table 3.

**Table 3:** Odds ratios in polytrauma patients with clavicular fractures and associated injuries

***Location***	***n***	***Odds ratio***	***KI***	***p***
*Thorax/chest*	74	4.5	1.32–15.30	0.016
*Arm/shoulder*	74	33.23	6.88–16.42	0.001
*Extremities*	74	12.52	1.5–102.94	0.004
*External*	74	9.17	2.7–30.90	>0.001

## Discussion

The number of people dying due to an accident continues to rise. While 18,527 cases were reported in Germany in 2007, 20,242 people died in 2010 [1]. There have been many attempts to prevent accidents, and the management of seriously injured patients has improved. Life-threatening injuries are typically found in the thoracic and abdominal area and in the long bones [2]. For this reason, these areas are the focus of attention initially. Therefor plain x-rays of the chest and pelvis as well as FAST (focus assessed sonography in trauma) is used in our institution. Nevertheless, overlooked and delayed diagnoses remain common problems in polytrauma patients. Pfeifer et al. reported that up to 22.3% of clinically significant injuries are missed in polytraumatized patients [3], although the reported rate of missed injuries varies widely (1.3–39%).

Injuries in the upper extremity region may not be immediately life threatening but they have a massive impact on limb survival, patient convalescence and rehabilitation. In all cases early diagnosis and correct treatment is important [7-11]. When focusing on the upper extremities, 33.3% [4] to 38.2% [5] of injuries are reported to have been missed. Guly distinguished between injuries of the wrist, hand and arm and reported rates of 17.2% (wrist), 21.7% (hand) and 15.1% (arm) of undetected injuries [6]. Others reported 4–8% (wrist/hand) and 11–12% (arm) [12,13] of missed injuries of the upper extremities. Aim of the present study was to find a co-incidence of easily diagnosed injuries that may help to identify concomitant trauma of the upper limbs. Although the emphasis is not specifically on missed injuries in this study, the found correlations help to focalize on additional injuries of the upper extremity region. The importance of treatment of upper limb injuries is obvious when the long-term results are considered. Stalp et al. reported that 16% of patients had moderate or severe restrictions after injuries of the upper extremities according to the Musculoskeletal Function Assessment [14]. Mkandawire et al. found that after 5 years patients with severe trauma (ISS > 15) and shoulder girdle injuries had persisting disability in 48% and chronic pain in 45% of cases. Functional problems with activities of daily living, work, sport and mobility were reported in up to 38% of patients. Persisting disability was seen in patients with fractures of the upper limb in 66% of cases and chronic pain was reported in 62% of these cases. Functional problems with activities of daily living, work, sport and mobility were reported in up to 66% of these patients. The author questioned whether earlier and better fixation and rehabilitation of fractures in severely injured patients might improve these results [15]. Although there is no literature about the optimum timing of surgical treatment for fractures of the upper extremities in multiply injured patients, shaft fractures should be surgically managed soon after diagnosis [16]. Tscherne et al. published a hierarchy of urgency for the operative treatment of fractures in the polytrauma patient. While treatment of upper extremity fractures follows management of the tibia, femur, pelvis and spine it precedes complex joint reconstructions, definitive treatment of maxillofacial injuries and soft tissue reconstruction [17]. As the multiply injured patient is always included in heterogeneous groups, there are no comparative studies that deal specifically with the most suitable operative procedure in fractures of the upper extremity [18-23].

Further studies have shown that missed injuries and delayed diagnoses cause significantly longer hospital stays (15.7–42.1 days vs. 7.9–26.7 days) in polytrauma patients. These patients also stayed in the intensive care unit for a prolonged period (5.4–10.9 days vs. 1.5–5.7 days) [4,5,24,25]. The present study supports these findings and underlines the importance of early diagnosis of all relevant injuries. High rates of mortality have been reported in polytrauma patients with missed injuries and there is evidence for a relationship between delayed diagnosis and morbidity [12].

The rate of missed injuries needs to be reduced. Clinical and radiological examinations remain of great importance. While it is easier to make the correct diagnosis in clinically alert patients, the examiner should be aware of missed injuries in unconscious and intubated patients. Here further diagnostic tools would be beneficial [26-28]. Some studies have revealed a lack of admission radiographs of the injured area as one reason for overlooked injuries and others have reported misinterpreted x-rays [29,30]. Clinical experience and assessment errors were also found to play an important role [4,5,13,31,32]. It is rare that just one factor leads to the lack of diagnosis. More commonly a combination of different factors is responsible for missing an injury [32]. As mentioned above a polytrauma patient with reduced consciousness or that is unconscious and intubated needs careful attention during the primary and secondary survey. A tertiary survey helps to determine initially overlooked injuries within 24 h of admission. Up to 90% of clinically significant injuries that were initially not diagnosed were found during a tertiary survey [32]. Although the timing of a tertiary survey may vary and the patient could be examined before leaving the intensive care unit [5], it is helpful to have a conscious patient for this survey. Algorithms in our department were adapted towards a tertiary survey. Even though a tertiary survey should be performed there is no doubt that any injury should be diagnosed and treated as soon as possible. Therefore it is helpful to have indicators for further injuries.

This study is limited by its retrospective design and the small number of polytraumatized patients with a clavicular fracture in a single center institution. However, our hypothesis that a clavicular fracture indicates the presence of further injuries that might be missed during a primary survey was supported. These findings are important as a clavicular fracture in a polytrauma patient directs attention to otherwise overlooked injuries of the upper extremities. In addition, patients with a clavicular fracture are more severely injured than patients without a clavicular fracture. Based on our data we conclude that a clavicular fracture can be seen as an indicator of injury in polytrauma patients. Although adapting the trauma algorithm on primary survey is not necessary we pay special attention to the upper limb region especially in secondary and tertiary survey when a clavicular fracture is found. Beside reevaluation of standard blood tests, a tertiary trauma survey should be performed on an alert patient that is able to express pain during clinical assessment and involve careful review of initial x-rays. Attention must be paid to nerval and vascular injuries as well as covered soft tissue and ligamentous lesions. Due to the fact that musculoskeletal injuries are usually missed until tertiary survey, an experienced orthopaedic surgeon must be involved by then.

Further studies must identify the specific injury patterns that are associated with fracture of the clavicle in polytraumatized patients.

## Advances in knowledge

This is the first study focusing on clavicular fracture as an indicator of further injuries in polytrauma patients.

## Competing interests

The authors declare that there are no conflicts of interest including financial, consultant, institutional and other relationships.

## Authors’ contribution

The work presented here involved the collaboration of all authors. KH, TD and MP defined the research theme and designed the methods, collected and analyzed the data, interpreted the results and wrote the paper. RP, FH and HCP worked on interpretation and discussed the analyses, interpretation, and presentation. HCP gave critical and final approval. All authors have contributed to, seen and approved the manuscript.
